# A Validation Approach of an End-to-End Whole Genome Sequencing Workflow for Source Tracking of *Listeria monocytogenes* and *Salmonella enterica*

**DOI:** 10.3389/fmicb.2018.00446

**Published:** 2018-03-14

**Authors:** Anne-Catherine Portmann, Coralie Fournier, Johan Gimonet, Catherine Ngom-Bru, Caroline Barretto, Leen Baert

**Affiliations:** ^1^Nestec Ltd., Nestle Research Center, Lausanne, Switzerland; ^2^Nestle Institute of Health Sciences SA, EPFL Innovation Park, Lausanne, Switzerland

**Keywords:** validation, SNP, WGS, *Listeria monocytogenes*, *Salmonella enterica*, phylogeny, source tracking, outbreak

## Abstract

Whole genome sequencing (WGS), using high throughput sequencing technology, reveals the complete sequence of the bacterial genome in a few days. WGS is increasingly being used for source tracking, pathogen surveillance and outbreak investigation due to its high discriminatory power. In the food industry, WGS used for source tracking is beneficial to support contamination investigations. Despite its increased use, no standards or guidelines are available today for the use of WGS in outbreak and/or trace-back investigations. Here we present a validation of our complete (end-to-end) WGS workflow for *Listeria monocytogenes* and *Salmonella enterica* including: subculture of isolates, DNA extraction, sequencing and bioinformatics analysis. This end-to-end WGS workflow was evaluated according to the following performance criteria: stability, repeatability, reproducibility, discriminatory power, and epidemiological concordance. The current study showed that few single nucleotide polymorphism (SNPs) were observed for *L. monocytogenes* and *S. enterica* when comparing genome sequences from five independent colonies from the first subculture and five independent colonies after the tenth subculture. Consequently, the stability of the WGS workflow for *L. monocytogenes* and *S. enterica* was demonstrated despite the few genomic variations that can occur during subculturing steps. Repeatability and reproducibility were also demonstrated. The WGS workflow was shown to have a high discriminatory power and has the ability to show genetic relatedness. Additionally, the WGS workflow was able to reproduce published outbreak investigation results, illustrating its capability of showing epidemiological concordance. The current study proposes a validation approach comprising all steps of a WGS workflow and demonstrates that the workflow can be applied to *L. monocytogenes* or *S. enterica*.

## Introduction

The use of Whole genome sequencing (WGS) for outbreak investigation and pathogen source tracking has been described in several publications since a few years (Leekitcharoenphon et al., 2014; Schmid et al., 2014; Octavia et al., 2015; Wuyts et al., 2015; Dallman et al., 2016; Jackson et al., 2016; Wilson et al., 2016; Inns et al., 2017). Current sequencing platforms (Illumina, Ion torrent, Oxford, PacBio) can be used and many bioinformatics approaches are available such as whole genome Multi Locus Sequence Typing (wgMLST), core genome MLST (cgMLST) or high quality Single Nucleotide Polymorphism (hqSNP). Several public health agencies and food authorities are using WGS for outbreak investigations or pathogen source tracking (Chen et al., 2017; Inns et al., 2017; Katz et al., 2017; Moran-Gilad, 2017), and food industries are gradually evaluating or implementing the WGS technology. In the food industry, WGS used for source tracking is beneficial to provide leads on the true cause of a contamination event, to find patterns and eventually prevent re-occurring issues. No standards or guidelines are available today on the use of WGS applied to outbreak and/or trace-back investigations. Working groups have been set up (e.g., International Life Sciences Institute, ILSI and Global Microbial Identifier, GMI) to address harmonization of methodologies and results interpretation. In addition, the development of a standard has started within the International Organization for Standardization (ISO TC34-SC9-WG25) and aims at developing an internationally harmonized WGS methodology for source tracking.

The objective of the current study was to validate the end-to-end WGS workflow used for source tracking of *Listeria monocytogenes* and *Salmonella enterica*. Validation is crucial as WGS is used as a typing method. Thus, it is important to ensure the reliability when similarities or differences between genomes are observed. Since WGS for source tracking is an analytical approach often carried out by multiple groups/laboratories of different expertise/technologies (microbiology, sequencing technologies, bioinformatics, and genomics), it is important to emphasize the validation should cover the end-to-end workflow including all steps. The end-to-end WGS workflow in the current study consisted of four steps: subculturing isolates to obtain pure colonies, extracting DNA, performing short read sequencing with MiSeq Illumina and carrying out bioinformatics analysis based on read mapping allowing identification of hqSNPs with the CFSAN SNP Pipeline. Even though the CFSAN SNP Pipeline was previously evaluated on its robustness and accuracy (Davis et al., 2015), the end-to-end workflow has never been fully validated. The validation was done following the recommendations of the members of the European Study Group on Epidemiological Markers (ESGEM) and of the European Society for Clinical Microbiology and Infectious Diseases (ESCMID) (Struelens, 1996). In order to validate the end-to-end workflow from isolate subculture to bioinformatics analysis, the following performance criteria were tested: stability, repeatability, reproducibility and discriminatory power. The bioinformatics analysis pipeline was used to validate the epidemiological concordance. The evaluation of the stability assesses that there is no impact of genomic variation that might occur during bacterial replications, on the complete WGS workflow ability to recognize the clonal relatedness of isolates. Repeatability evaluates the impact of reiterating all the steps of the workflow by the same operators (sequencing of isolates extracted during the same DNA extraction, and sequencing run). Reproducibility evaluates the impact of different operators on DNA extraction and on sequencing, with different sequencing instruments and in different laboratories. The purpose of assessing the discriminatory power is to ensure that unrelated strains can be differentiated while revealing relatedness of clonal isolates. The epidemiological concordance evaluates the capability of the bioinformatics part of the WGS workflow to reproduce results from previous published outbreak investigations that used different bioinformatics software. In addition, the impact of using PacBio contigs or Illumina contigs as reference for the read mapping was evaluated.

The current study proposes a validation approach comprising all steps of a WGS workflow. This study presents results on performance characteristics applied to our complete WGS workflow used for source tracking of *L. monocytogenes* and *S. enterica*.

## Materials and methods

### Bacteria and subculture conditions

*Listeria monocytogenes* and *Salmonella enterica* strains (Table 1) were stored on cryobeads (TSC, Lancashire, UK) at −80°C. Strains were streaked on Trypcase Soy Agar (TSA) (BioMérieux, Marcy l'Etoile, France) to obtain single colonies. After 24 ± 2 h incubation at 37°C, one colony was used to inoculate 4 mL Brain Heart Infusion (BHI) (Thermo Scientific™ Oxoid™, Hampshire, UK) and was incubated at 37°C for 6–8 h. One mL of inoculated BHI was taken and centrifuged (5,000 × g, 5 min). The pellet was stored at −20°C until the DNA extraction was performed.

**Table 1:** Stability and Repeatability dataset for *L. monocytogenes* and *S. enterica*.

**Strain ID**	**Biosample**	**PacBio accession number**	**Genus/species**	**Serotype**
PIR00542	SAMN08125517	CP025222	*L. monocytogenes*	1/2a
PIR00543	SAMN08125518	CP025221		1/2a
PIR00545	SAMN08125520	CP025560, CP025561		1/2b
PIR00540	SAMN08125515	CP025568, CP025569		1/2c
PIR00541	SAMN08125516	CP025566, CP025567		1/2c
PIR00544	SAMN08125519	CP025562, CP025563, CP025564, CP025565		4b
PIR00546	SAMN08125521	CP025220		4b
PIR00547	SAMN08125522	CP025219		4b
PIR00532	SAMN08125525	CP025557, CP025558, CP025559	*S. enterica*	Enteritidis
PIR00558	SAMN08125532	CP025553, CP025554		Enteritidis
PIR00533	SAMN08125526	PKPH00000000		Hadar
PIR00534	SAMN08125527	PKPG00000000		Hadar
PIR00537	SAMN08125530	CP025217		Tennessee
PIR00535	SAMN08125528	CP025218		Tennessee
PIR00536	SAMN08125529	CP009102		Typhimurium
PIR00538	SAMN08125531	CP025555, CP025556		Typhimurium

### DNA extraction

#### DNA extraction for short read sequencing: illumina MiSeq and HiSeq

##### Listeria monocytogenes

The bacterial pellet was suspended in 160 μL buffer P1 (Qiagen, Hilden, Germany). Twenty microliter of lysozyme from chicken egg solution (Sigma, 200 mg/mL in DNA free water) was added and incubated at 37°C with agitation at 900 rpm for 2 h (Eppendorf ThermoMixer C). Proteinase K (20 μL; Qiagen) was added and the solution was incubated 1 h at 56°C. The QIAamp DNA Mini kit was used according to the supplier's instructions. DNA was eluted in 100 μL Elution buffer (Qiagen) and stored at −20°C until further analysis.

##### Salmonella enterica

The bacterial pellet was suspended in 180 μL Buffer ATL (Qiagen). Proteinase K (20 μL; Qiagen) was added and the solution was incubated 1 h at 56°C. The QIAamp DNA Mini kit was used according to the supplier's instructions. DNA was eluted in 100 μL Elution buffer (Qiagen) and stored at −20°C until further analysis.

The Qubit™ dsDNA HS Assay kit (Invitrogen) was used to measure the DNA concentration with the Qubit® 2.0 Fluorometer (Invitrogen) according to the supplier's instructions. DNA was standardized to 10 ng/μL with Elution buffer (Qiagen) and stored at −20°C until used for sequencing.

#### DNA extraction for long read sequencing: pacific biosciences (PacBio)

The Gentra Puregene Yeast/Bact. kit (Qiagen) was used for the DNA purification of bacterial cultures.

##### Listeria monocytogenes

One colony (see section Bacteria and Subculture Conditions) was used to inoculate BHI broth and grown at 37°C overnight. Five mL of broth were centrifuged (3,500 rpm, 5 min) and supernatant was discarded. The pellet was washed three times using 1 mL of Phosphate-Buffered Saline (PBS). DNA extraction was carried out following supplier's instructions indicated in the following protocol: DNA purification from Gram-Positive bacteria.

##### Salmonella enterica

One colony (see section Bacteria and Subculture Conditions) was used to inoculate BHI broth and grown at 37°C until mid-exponential phase (4–5 h incubation). Five milliliter of broth were centrifuged (3,500 rpm, 5 min) and supernatant was discarded. The pellet was washed three times using 1 mL of PBS. DNA extraction was carried out following supplier's instructions indicated in the following protocol: DNA purification from Gram-Negative bacteria.

DNA was stored at −20°C until further analysis. The Qubit™ dsDNA BR Assay kit (Invitrogen) was used to measure the DNA concentration with the Qubit® 2.0 Fluorometer (Invitrogen) according to the supplier's instructions.

### Sequencing

#### Short read sequencing (illumina MiSeq and HiSeq)

DNA was normalized at 0.2 ng/μL in order to start with 1 ng to perform a sequencing library preparation using Nextera XT kit (Illumina) following the supplier's instructions. A final AMPure beads purification at ratio 0.6 was performed on a Sciclone robotic platform from Perkin Elmer. The quality and quantity of each library were evaluated using a capillary electrophoresis method (LabChip GX Touch from Perkin Elmer). Libraries were pooled based on molarity calculated by the LabChip GX Touch. The equimolar pool was assembled using a Hamilton robotic platform.

##### MiSeq

The sequencing was performed on a MiSeq platform (Illumina) using v2 chemistry, for a 2 × 250 cycles run. The pool was spiked with 2% PhiX, loaded at 12 pM and 16 to 19 samples were loaded per MiSeq flow cell (corresponding to ~2 million reads pass filter per sample).

##### HiSeq

To ensure each library was present in the pool before sequencing, the equimolar pool was controlled by a MiSeq run v2 chemistry for 2 × 20 cycles. Then the pool was sequenced on a HiSeq 2500 platform (Illumina) using Rapid v2 chemistry. The pool was spiked with 2% PhiX, loaded between 9 or 12 pM and respectively 190 or 84 samples were loaded per HiSeq flow cell (corresponding to ~3 or ~9 million reads pass filter per sample).

All sequences have been submitted to the National Center for Biotechnology Information (NCBI) in BioProject: PRJNA420913 and all sequence read archive numbers (SRR) are available in Tables 2–**6**.

**Table 2:** *S. enterica* and *L. monocytogenes* sequences dataset and their Sequence Read Archive numbers used for the stability and repeatability experiments^a^.

**Strain ID**	**Isolates from independent colonies of the first subculture**					**Isolates from independent colonies of the tenth subculture**				
	**A**	**B**	**C**	**D**	**E**	**A1**	**A2**	**A3**	**A4**	**A5**
PIR00540	SRR6347524	SRR6347523	SRR6347352	SRR6347349	SRR6347348	SRR6347347	SRR6347542	SRR6347538	SRR6347486	SRR6347426
PIR00541	SRR6347510	SRR6347509	SRR6347512	SRR6347511	SRR6347506	SRR6347505	SRR6347508	SRR6347507	SRR6347514	SRR6347513
PIR00542	SRR6347487	SRR6347488	SRR6347489	SRR6347490	SRR6347491	SRR6347492	SRR6347493	SRR6347494	SRR6347484	SRR6347485
PIR00543	SRR6347473	SRR6347472	SRR6347471	SRR6347470	SRR6347469	SRR6347468	SRR6347467	SRR6347466	SRR6347465	SRR6347464
PIR00544	SRR6347427	SRR6347432	SRR6347424	SRR6347425	SRR6347442	SRR6347443	SRR6347403	SRR6347441	SRR6347428	SRR6347429
PIR00545	SRR6347407	SRR6347406	SRR6347409	SRR6347408	SRR6347411	SRR6347410	SRR6347413	SRR6347412	SRR6347405	SRR6347404
PIR00546	SRR6347388	SRR6347389	SRR6347390	SRR6347391	SRR6347384	SRR6347385	SRR6347386	SRR6347387	SRR6347382	SRR6347383
PIR00547	SRR6347353	SRR6347402	SRR6347351	SRR6347350	SRR6347357	SRR6347356	SRR6347355	SRR6347354	SRR6347360	SRR6347359
PIR00532	SRR6347380	SRR6347381	SRR6347378	SRR6347379	SRR6347376	SRR6347377	SRR6347374	SRR6347375	SRR6347372	SRR6347373
PIR00533	SRR6347545	SRR6347544	SRR6347547	SRR6347546	SRR6347541	SRR6347540	SRR6347543	SRR6347527	SRR6347539	SRR6347525
PIR00534	SRR6347478	SRR6347479	SRR6347480	SRR6347481	SRR6347474	SRR6347475	SRR6347476	SRR6347477	SRR6347482	SRR6347483
PIR00535	SRR6347498	SRR6347497	SRR6347496	SRR6347495	SRR6347502	SRR6347501	SRR6347500	SRR6347499	SRR6347504	SRR6347503
PIR00536	SRR6347358	SRR6347361	SRR6347521	SRR6347522	SRR6347519	SRR6347520	SRR6347517	SRR6347518	SRR6347515	SRR6347516
PIR00537	SRR6347535	SRR6347534	SRR6347537	SRR6347536	SRR6347531	SRR6347530	SRR6347533	SRR6347532	SRR6347529	SRR6347528
PIR00538	SRR6347364	SRR6347365	SRR6347366	SRR6347367	SRR6347368	SRR6347369	SRR6347370	SRR6347371	SRR6347362	SRR6347363
PIR00558	SRR6347399	SRR6347398	SRR6347397	SRR6347396	SRR6347395	SRR6347394	SRR6347393	SRR6347392	SRR6347401	SRR6347400
Stability^b^	x	x	x	x	x	x	x	x	x	x
Repeatability^b^	x	x	x	x	x					

a *For each strain ten genome sequences were generated: five from independent colonies of the first subculture (A, B, C, D, and E) and five from independent colonies after the tenth subculture (A1, A2, A3, A4, and A5). For each analysis the de novo assembled Illumina reads of isolate (A) were used as reference*.

b *×:used in the experimental setup*.

#### Long read sequencing (pacific biosciences)

High molecular weight DNA was sheared using g-TUBE (Covaris) to obtain around 20 kb DNA fragments. After shearing, the DNA size distribution was checked using the TapeStation2500 system (Agilent) or Fragment Analyzer (Advanced Analytical).

DNA was quantified using the Qubit system (Qubit 2.0 Fluorometer and dsDNA Assay HS) and around 5 μg of the sheared DNA was used to prepare a SMRTbell library following the protocol *Procedure and Checklist* −*20 kb Template Preparation Using BluePippin*™ *Size-Selection System*. (Pacific Biosciences). The library was size selected using a BluePippin system (Sage Science). Due to low DNA quantity after shearing for isolates PIR00544 and PIR00547, the 10–20 kb Template Preparation and Sequencing with Low-Input DNA protocol (Pacific Biosciences) was applied without size selection.

The library was sequenced using RSII platform (Pacific Biosciences) on one or two SMRT cells with P6-C4 chemistry and MagBeads loading (Pacific Biosciences). Sequencing time was between 240 and 380 min.

All sequences accession numbers are available in Table 1 (BioProject: PRJNA420913).

### Bioinformatics analysis

#### Raw data quality check

In order to evaluate the sequencing run quality, fastq files were assessed with the FastQC software (v0.11.5) (http://www.bioinformatics.babraham.ac.uk/projects/fastqc/). To choose the best isolate for genome assembly that will serve as reference during hqSNP analysis, the reads should pass the “per base sequence quality” FASTQC threshold.

#### Genome assembly

Illumina reads were first trimmed with Trimmomatic software (version 0.36) (Bolger et al., 2014). Bases at both ends of reads generated with Illumina were removed within a sliding window of 10 base pairs when the average quality in this window was lower than Q20 score (equivalent Phred score). Then, SPAdes (v3.9.0) (Bankevich et al., 2012) *de novo* assembled the trimmed reads and generated a multi contigs genome.

The PacBio genomes were built with a *de novo* assembly carried out using the PacBio Hierarchical Genome Assembly Process (HGAP) version 3 (Pacific Biosciences), closure of the genome by Circlator (Hunt et al., 2015) and final polishing by Quiver v1 (Pacific Biosciences).

#### Single nucleotide polymorphism (SNP) analysis

High quality SNP pipeline developed by the Center for Food Safety and Applied Nutrition (CFSAN SNP Pipeline v.1.0.0/FDA) was used for SNP calling on *L. monocytogenes* and *S. enterica* isolates (Davis et al., 2015). The reference genome was either a genome generated by PacBio assembly, a public available genome or a genome assembly from Illumina reads.

#### Phylogenetic analysis

Maximum-likelihood phylogenetic trees were built with GARLI (Version 2.01.1067) on the SNP analysis result and visualized with Figtree (version 1.4.3) (http://tree.bio.ed.ac.uk/software/figtree/). All trees shown in this paper are midpoint rooted.

### Experimental design for performance criteria

#### Stability

*Listeria monocytogenes* and *S. enterica* strains from −80°C were streaked on TSA and Columbia agar (BioMérieux) respectively, for 24 ± 2 h at 37°C to obtain single colonies. One colony was dissolved in 0.5 mL tryptone (1 g/L; Oxoid) salt (8.5 g/L; Merck, Darmstadt, Germany) solution streaked on Columbia agar (*L. monocytogenes*) or TSA (*S. enterica*), and incubated for 24 ± 2 h at 37°C to ensure colonies were pure. This procedure was repeated three times. Afterwards, five colonies (A, B, C, D, and E) were picked from the same agar plate and each colony was grown in 4 mL BHI broth as indicated in section Bacteria and Subculture Conditions. DNA was extracted as described in section DNA Extraction and sequenced as described in section Sequencing. In addition, colony A was subcultured 10 times by emulsifying one colony in tryptone salt solution, streaking on TSA/Columbia agar and incubating for 24 ± 2 h at 37°C. This procedure was repeated 10 times. After 10 subcultures, five colonies (A1, A2, A3, A4, and A5) were selected and each colony was grown in 4 mL BHI broth as indicated in section Bacteria and Subculture Conditions. DNA was extracted as described in section DNA Extraction and sequenced as described in section Sequencing. The dataset used to evaluate stability is presented in Tables 1, 2. SNP analysis was carried out using for each isolate two reference genomes, one created by PacBio sequencing and one by using the contigs after assembling HiSeq sequence reads obtained from colony A.

#### Repeatability

The repeatability was evaluated using the sequences obtained from the eight *L. monocytogenes* and eight *S. enterica* from the stability experiment (colonies A, B, C, D, and E as described in Table 2). DNA was extracted the same day by the same operator and isolates were sequenced on the same HiSeq run with the same library preparation.

SNP analysis was carried out using for each isolate two reference genomes, one created by PacBio sequencing and one by using the contigs after assembling HiSeq sequence reads obtained from colony A.

#### Reproducibility

*Listeria monocytogenes* strains PIR00542, PIR00544 and PIR00547 and *S. enterica* strains PIR00534, PIR00536 and PIR00503 were selected to perform the reproducibility experiment. DNA extractions of each set of isolates were carried out by two operators. DNA obtained by each operator was split in three tubes per isolate and sequenced on different Illumina MiSeq instruments using different batches of V2 chemistry by three different operators in two different laboratories. A total of 18 genomes sequences per species was analyzed.

SNP analysis was carried out using for each isolate two reference genomes, one created by PacBio sequencing and one by using the contigs after assembling MiSeq sequence reads (Supplementary Materials reproducibility table). The dataset used to evaluate reproducibility is presented in Tables 3, 4.

**Table 3:** *L. monocytogenes* sequence data used for assessing the reproducibility by hqSNP analysis.

**Strain ID**	**Sequence Read Archive number**	**DNA extraction operator (DEO)^a^**	**Sequencing operator (SO)^b^**	**Sequencing laboratories (SL)^c^**	**Accession number for PacBio sequence used as reference^d^**
PIR00542	^*^SRR6347440	DEO1	SO1	SL1	CP025222
	SRR6347433	DEO1	SO2	SL1	
	SRR6347434	DEO1	SO3	SL2	
	SRR6347435	DEO2	SO1	SL1	
	SRR6347436	DEO2	SO2	SL1	
	SRR6347430	DEO2	SO3	SL2	
PIR00544	^*^SRR6347431	DEO1	SO1	SL1	CP025562, CP025563, CP025564, CP025565
	SRR6347459	DEO1	SO2	SL1	
	SRR6347458	DEO1	SO3	SL2	
	SRR6347457	DEO2	SO1	SL1	
	SRR6347456	DEO2	SO2	SL1	
	SRR6347463	DEO2	SO3	SL2	
PIR00547	^*^SRR6347462	DEO1	SO1	SL1	CP025219
	SRR6347461	DEO1	SO2	SL1	
	SRR6347460	DEO1	SO3	SL2	
	SRR6347455	DEO2	SO1	SL1	
	SRR6347454	DEO2	SO2	SL1	
	SRR6347526	DEO2	SO3	SL2	

a *Two different operators performed the DNA extractions (DEO1 and DEO2)*.

b Three different sequencing operators (SO1, SO2, and SO3) sequenced the DNA at

c *two different sequencing laboratories (SL1 and SL2)*.

d For each analysis two references were used and evaluated: de novo assembled Illumina reads of isolate (^*^) and PacBio contigs.

**Table 4:** *S. enterica* sequence data used for assessing the reproducibility by hqSNP analysis.

**Isolate ID**	**Sequence Read Archive number**	**DNA extraction operator (DEO)^a^**	**Sequencing operator (SO)^b^**	**Sequencing laboratories (SL)^c^**	**Accession number for PacBio sequence used as reference^d^**
PIR00534	^*^SRR6347416	DEO1	SO1	SL1	PKPG00000000
	SRR6347417	DEO1	SO2	SL1	
	SRR6347414	DEO1	SO3	SL2	
	SRR6347415	DEO2	SO1	SL1	
	SRR6347420	DEO2	SO2	SL1	
	SRR6347421	DEO2	SO3	SL2	
PIR00536	^*^SRR6347418	DEO1	SO1	SL1	CP009102.1
	SRR6347419	DEO1	SO2	SL1	
	SRR6347422	DEO1	SO3	SL2	
	SRR6347423	DEO2	SO1	SL1	
	SRR6347445	DEO2	SO2	SL1	
	SRR6347444	DEO2	SO3	SL2	
PIR00503	^*^SRR6347447	DEO1	SO1	SL1	PKPF00000000
	SRR6347446	DEO1	SO2	SL1	
	SRR6347449	DEO1	SO3	SL2	
	SRR6347448	DEO2	SO1	SL1	
	SRR6347451	DEO2	SO2	SL1	
	SRR6347450	DEO2	SO3	SL2	

a *Two different operators performed the DNA extractions (DEO1 and DEO2)*.

b Three different sequencing operators (SO1, SO2, and SO3) sequenced the DNA at

c *two different sequencing laboratories (SL1 and SL2)*.

d For each analysis two references were used and evaluated: de novo assembled Illumina reads of isolate (^*^) and PacBio contigs.

#### Discriminatory power

Since the WGS method has a high resolution capability, not only isolates known to be unrelated (derived from the information provided by bacterial reference collections of the selected strains), but also isolates having clonal relatedness were included in the analysis in order to show that the end-to-end workflow was able to discriminate correctly while not missing similarities.

The choice of *L. monocytogenes* strains to be included in the discriminatory power experiment was based on serotype selection (4b, 1/2a, and 1/2c). For serotype 4b, two strains known to be unrelated were selected (Table 5). With this design, four distinct groups of genomes were supposed to be discriminated while not missing similarities within groups. Table 5 summarizes the different groups of related *L. monocytogenes* isolates.

**Table 5:** *L. monocytogenes* sequence dataset for assessing the discriminatory power by hqSNP analysis.

**Serotype**	**Epidemiological relationship**	**Isolate ID**	**SRR number**	**Groups with clonal relationship**
1/2c	ATCC 51779 (Belgium) Independent colonies from the first subculture	PIR00541 (A)	SRR6347510	Group 1
		PIR00541 (B)	SRR6347509	
		PIR00541 (C)	SRR6347512	
1/2a	ATCC 51775 (Belgium) Independent colonies from the first subculture	PIR00542 (A)	SRR6347487	Group 2
		PIR00542 (B)	SRR6347488	
		PIR00542 (C)	SRR6347489	
4b	ATCC 13932 (Germany) Independent colonies from the first subculture	PIR00547 (A)	SRR6347353	Group 3
		PIR00547 (B)	SRR6347402	
		PIR00547 (C)	SRR6347351	
	NCTC 11994 (UK)	PIR00492^a^	SRR6347453	Group 4
			SRR6347452	
		PIR00548^b^	SRR6347437	
			SRR6347438	
			SRR6347439	

a *For this isolate two genome sequences were generated from independent subcultures*.

b *For this isolate three genome sequences were generated from independent subcultures*.

Groups 1, 2, and 3 were respectively composed of sequences from independent colonies A, B, and C coming from three different strains of the stability experiment. Group 4 was composed of two NCTC 11994 isolates bought from BioMérieux at two different dates (2011: PIR00492 and 2015: PIR00548).

For *S. enterica*, three serovars (Enteritidis, Typhimurium and Tennessee) were selected to perform three distinct discriminatory experiments. For each serovar, strains were selected based on the epidemiological relationships information, in order to create three to four groups to be discriminated while not missing similarities within groups. Table 6 summarizes the groups per serovar. For the three serovars, groups 1 and 2 were both composed of sequences obtained from three independent colonies (A, B, and C) coming from two different strains of the stability experiment.

**Table 6:** *S. enterica* sequence dataset for assessing the discriminatory power by hqSNP analysis.

**Serovars**	**Isolate ID**	**Sequence Read Archive numbers**	**Epidemiological relationship**	**Groups with clonal relationship**
*S*. Enteritidis	PIR00532 (A)	SRR6347380	Independent colonies from the first subculture^a^	Group 1
	PIR00532 (B)	SRR6347381		
	PIR00532 (C)	SRR6347378		
	PIR00558 (A)	SRR6347399	ATCC BAA-708 Independent colonies from the first subculture	Group 2
	PIR00558 (B)	SRR6347398		
	PIR00558 (C)	SRR6347397		
	PHE: 32476	SRR1965122	European egg outbreak	Group 3
	PHE: 21785	SRR1965313		
	PHE: 32477	SRR1966289		
	S14BD01753	SRR2088895	Belgian egg outbreak	Group 4
	S14FP01877	SRR2088898		
*S*. Tennessee	PIR00535 (A)	SRR6347498	ATCC 10722 Independent colonies from the first subculture	Group 1
	PIR00535 (B)	SRR6347497		
	PIR00535 (C)	SRR6347496		
	PIR00537 (A)	SRR6347535	Independent colonies from the first subculture	Group 2
	PIR00537 (B)	SRR6347534		
	PIR00537 (C)	SRR6347537		
	CFSAN001387	SRR965704	American peanut butter outbreaks	Group 3
	CFSAN001349	SRR1177176		
*S*. Typhimurium	PIR00536 (A)	SRR6347358	ATCC13311 Independent colonies from the first subculture	Group 1
	PIR00536 (B)	SRR6347361		
	PIR00536 (C)	SRR6347521		
	PIR00538 (A)	SRR6347364	Independent colonies from the first subculture	Group 2
	PIR00538 (B)	SRR6347365		
	PIR00538 (C)	SRR6347366		
	Styph-0803T57157	ERR277220	Large Denmark outbreak	Group 3
	Styph-0808S61603	ERR277226		
	1687	SRR1645473	Australian chocolate mousse outbreak	Group 4
	1700	SRR1645486		

a *Sequence data obtained from the stability experiment described in Table 2*.

For *S*. Enteritidis, group 3 was composed of three related isolates from an European egg related outbreak (Dallman et al., 2016). Group 4 was composed of two sequenced isolates from a Belgian egg related outbreak published by Wuyts and coworkers (Wuyts et al., 2015).

For *S*. Tennessee, group 3 was composed of two sequenced isolates from a peanut butter outbreak in USA (2006–2007) published in 2016 by CFSAN FDA (Wilson et al., 2016).

For *S*. Typhimurium, group 3 was composed of two sequenced isolates from a large Denmark outbreak (Leekitcharoenphon et al., 2014). Group 4 was composed of two sequenced isolates from an Australian chocolate mousse outbreak (Octavia et al., 2015).

#### Epidemiological concordance

Struelens and coworkers indicated that the epidemiological concordance should be used to verify that the method is able to obtain the same conclusions as the ones from well-defined outbreak studies (Struelens, 1996). In this study, one published outbreak of *L. monocytogenes* and one of *S. enterica* were analyzed and the outcomes of the bioinformatics analysis were compared to the original findings. For each outbreak, the analysis was done following two approaches. In the first approach, the analysis was performed using the isolate reference of the authors with the CFSAN SNP Pipeline which is part of our workflow. Then, in a second approach, the analysis was reproduced using the food isolate as reference.

## Results

### Stability

The stability of *L. monocytogenes* and *S. enterica* was evaluated by the selection of eight *L. monocytogenes* and eight *S. enterica* strains. The selection of strains covered the major serotypes related to food. For each strain ten genome sequences were generated: five from independent colonies of the first subculture (A, B, C, D, and E) and five from independent colonies after the tenth subculture (A1, A2, A3, A4, and A5). The number of SNPs was determined between the 10 generated genome sequences per strain.

Table 7 summarizes the results of the hqSNP analysis for *L. monocytogenes*. For *L. monocytogenes* PIR00540, PIR00542, PIR00543, PIR00545, PIR00546, and PIR00547, no SNP was found between colonies of the first subculture (A, B, C, D, and E) and after the tenth subculture (A1, A2, A3, A4, and A5). For two strains, PIR00541 and PIR00544, few SNPs were identified. For PIR00541, colony B had 1 SNP compared to colonies A, C, D, and E. Colonies A, C, D, and E had 0 SNPs between them. After the tenth subculture, colonies A1, A2, A3, A4, and A5 acquired 1 SNP compared to colony A. However, this acquired SNP was different from the one from colony B. For PIR00544, no SNPs was found between colonies A, B, C, D, and E, but after the tenth subculture a maximum of 3 SNPs were identified for two colonies out of the five.

**Table 7:** Maximum SNPs observed during the stability test of *L. monocytogenes* between five independent colonies from the first subculture and after the tenth subculture.

**Isolate ID**	**Serotype**	**Max. SNPs between colonieswithin passage(A vs. B vs. C vs. D vs. E)(A1 vs. A2 vs. A3 vs. A4 vs. A5)**	**Max. SNPs after 10 subcultures(A, B, C, D, E) vs. (A1, A2, A3, A4, A5)**
PIR00542	1/2a	0	0
PIR00543	1/2a	0	0
PIR00545	1/2b	0	0
PIR00540	1/2c	0	0
PIR00541	1/2c	1	1
PIR00544	4b	2	3
PIR00546	4b	0	0
PIR00547	4b	0	0

Table 8 summarizes the results of the hqSNP analysis for *S. enterica* per serovar. For *S*. Hadar, no SNP was found between all colonies for the two strains PIR00533 and PIR00534. No SNP was found between all colonies for *S*. Typhimurium strain PIR00536. The hqSNP analysis of sequences obtained from strain PIR00538 revealed that colony A2 had 1 SNP compared to all the others colonies (A, B, C, D, E, A1, A3, A4, and A5). For *S*. Enteritidis, no SNP was found between all colonies for strain PIR00532. Colonies A1, A2, A3, A4, and A5 of strain PIR00558 had 1 SNP compared to the colonies from the first subculture (A, B, C, D, and E). Finally, for *S*. Tennessee, no SNP was found between all colonies for strain PIR00537, while PIR00535 colonies A1, A2, A3, A4, and A5, after the tenth subculture, had 1 SNP compared to the colonies from the first subculture (A, B, C, D, and E).

**Table 8:** Maximum SNPs observed during the stability test of *Salmonella enterica* between five independent colonies from the first subculture and after the tenth subculture.

**Isolate ID**	**Serovar**	**Max. SNPs between colonieswithin passage(A vs. B vs. C vs. D vs. E)(A1 vs. A2 vs. A3 vs. A4 vs. A5)**	**Max. SNPs after 10 subcultures(A, B, C, D, E) vs. (A1, A2, A3, A4, A5)**
PIR00532	Enteritidis	0	0
PIR00558	Enteritidis	0	1
PIR00533	Hadar	0	0
PIR00534	Hadar	0	0
PIR00537	Tennessee	0	0
PIR00535	Tennessee	0	1
PIR00536	Typhimurium	0	0
PIR00538	Typhimurium	0	1

### Repeatability

The sequence data used were selected from the stability experiment (colonies A, B, C, D, and E as described in Tables 1, 2) were used to assess the repeatability. In total 80 sequences from eight *L. monocytogenes* strains and eight *S. enterica* strains were analyzed.

Table 7 summarizes the results obtained for *L. monocytogenes*. No SNP was found between the five independent colonies (A, B, C, D, and E) for seven strains out of eight. For PIR00541, 1 SNP was observed in colony B compared to colonies A, C, D, and E from the first subculture, and after the tenth subculture another SNP was acquired. Knowing from the stability experiment that this strain acquired a random SNP, we assume that this acquisition was not due to the repeatability testing.

Table 8 summarizes the results obtained for *S. enterica*. For the eight tested *S. enterica* strains, no SNP was found between the five independent colonies (A, B, C, D, and E).

### Reproducibility

A total of 18 *L. monocytogenes* genome sequences and 18 *S. enterica* genome sequences were analyzed. For *L. monocytogenes* and *S*. Typhimurium and *S*. Hadar, no SNP was found between genome sequences obtained from different laboratories, operators and instruments.

### Discriminatory power

The results obtained for the discriminatory power evaluation of *L. monocytogenes* and *Salmonella enterica*, were in agreement with the design (Tables 5, 6).

*Listeria monocytogenes* strains of different serotypes were discriminated with more than 30,000 SNPs between groups, as well as strains of serotype 4b with more than 8,000 SNPs. Isolates within a group had a maximum of 1 SNP difference.

For *S*. Enteritidis, *S*. Tennessee, and *S*. Typhimurium, hqSNP analysis was performed per serotype. For the three serovars, results were in accordance with the design (Table 6). For *S*. Enteritidis, the groups were discriminated with more than 100 SNPs and isolates within a group had a maximum of 4 SNPs difference. The analysis of *S*. Tennessee isolates revealed more than 80 SNPs between the groups. Isolates within a group had a maximum of 5 SNPs difference. For the last serotype *S*. Typhimurium, the groups were discriminated with more than 490 SNPs and isolates within a group had a maximum of 3 SNPs.

### Epidemiological concordance

Jackson and coworkers reported the analysis of a *L. monocytogenes* outbreak presumably involving one patient from Ohio who had consumed a contaminated prepackaged romaine lettuce (Jackson et al., 2016). The Public Health Agency of Canada (PHAC) used Single Nucleotide Variant Phylogenomic pipeline (Petkau et al., 2017). While the Center for Disease Control and Prevention (CDC) used hqSNP (Lyve-SET; Katz et al., 2017) and wgMLST (BioNumerics, Applied Maths) to analyze this case. Twenty-six additional patient isolates, indistinguishable by pulsed-field gel electrophoresis (PFGE) analysis, were added to this investigation. Only the patient isolate from Ohio was found closely related to the prepackaged lettuce (PHAC: 0 SNP; CDC: 5 SNPs) and the other patient isolates were discriminated from this food contamination (CDC: ≥30 SNPs and ≥30 alleles). Authors concluded that only a single patient was likely associated with the lettuce recall.

**Figure 1:**
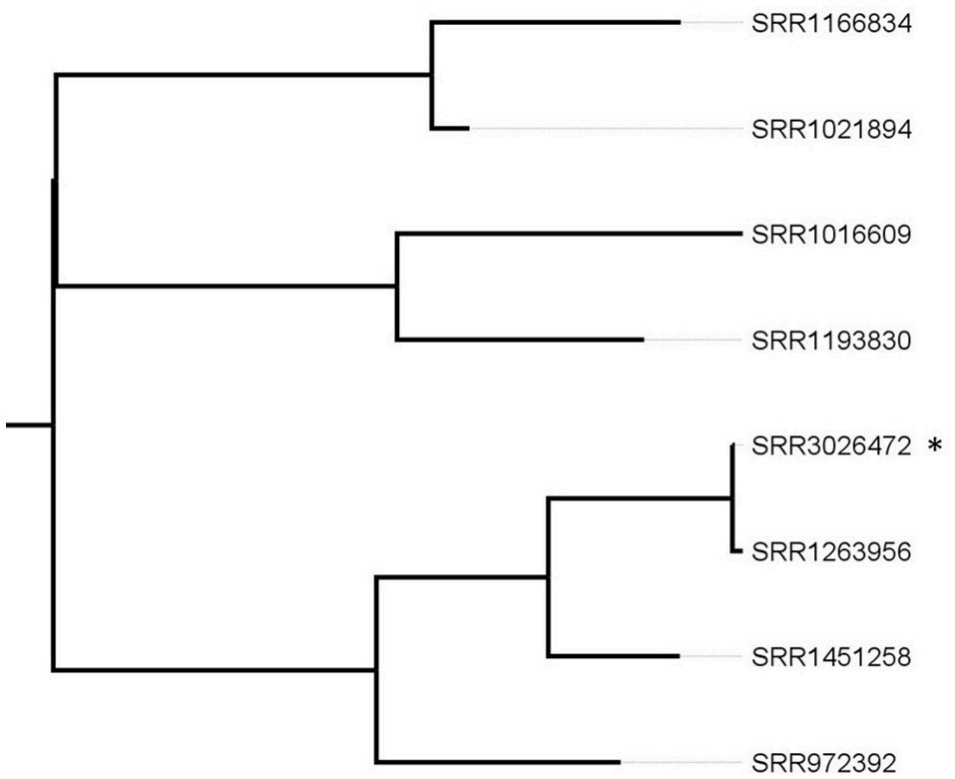
Phylogenetic tree based on SNP differences from selected patient isolates and the lettuce reference isolate. ^*^corresponds to the recalled lettuce isolate. The SRR1263956 corresponds to the Ohio patient isolate. Tree was generated with Garli and drawn with Figtree.

In a first approach, we repeated the analysis on the 26 patient isolates having the same PFGE profile, the Ohio patient and the lettuce isolate using the same reference as the authors (PNUSAL000564, SRR1166850) using the CFSAN SNP pipeline. This reference allowed to group together the Ohio patient isolate (SRR1263956) with the Canadian lettuce isolate (SRR3026472) and six additional patient isolates (SRR1016609, SRR1021894, SRR1166834, SRR1193830, SRR1451258, and SRR972392). All the other isolates had ≥100 SNPs and were not considered genetically linked to this outbreak. Since a distantly related reference genome can result in an underestimation of the genetic relatedness of the isolates being investigated, the group of eight isolates was analyzed separately. To analyze in more details the group composed of eight isolates with the CFSAN SNP Pipeline, the reads from the Canadian lettuce isolate were assembled to obtain a reference genome. With our analysis, 1 SNP was found between the sequence from the Ohio patient and the prepackaged romaine lettuce. SRR1451258 and SRR972392 had 28 and 51 SNPs and all the other isolates had ≥80 SNPs with the lettuce isolate, respectively (Figure 1). With this analysis, we reproduced the clustering of the Ohio patient isolate with the prepackaged lettuce isolate.

**Figure 2:**
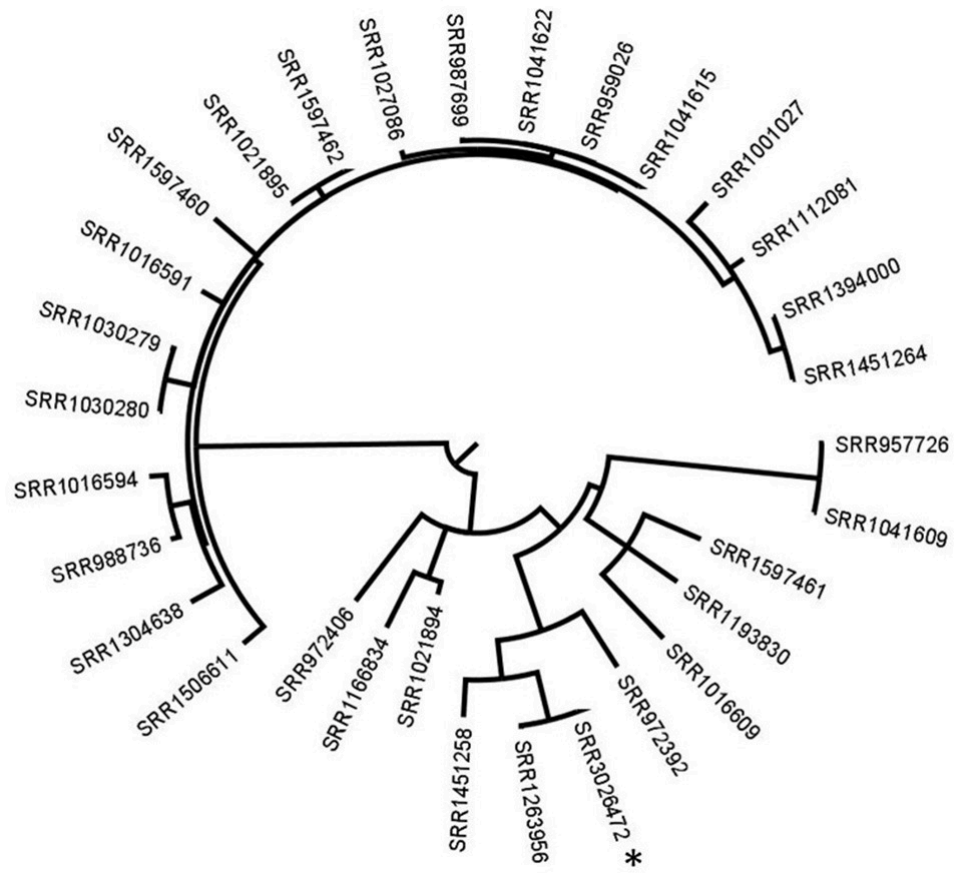
Phylogenetic tree based on SNP differences between all patient isolates and the reference lettuce isolate. ^*^corresponds to the recalled lettuce isolate. All the other isolates are patients' isolates. The SRR1263956 corresponds to the Ohio patient isolate. Tree was generated with Garli and drawn with Figtree.

In case no PFGE profile data are available and to conclude if any patient isolate is related to the food, the lettuce isolate would be our preferred reference to use with our methodology. Therefore, in the second approach, analysis was performed on the 26 patients isolates, the Ohio isolate and the lettuce isolate. The Ohio patient isolate grouped with the lettuce isolate with 1 SNP difference. SRR1451258 and SRR972392 had 28 and 51 SNPs respectively, and all the other isolates had ≥75 SNPs with the lettuce isolate (Figure 2).

In England and Wales, the national reference laboratory uses WGS with hqSNP analysis routinely, since April 2015, on all cultures of *Salmonella* sp. referred by local laboratories (Inns et al., 2017). In May 2015, they found a first cluster of 29 *S*. Enteritidis cases. In the following months, this cluster increased and Public Health England (PHE) declared an outbreak. The outbreak control team was in charge of finding the source of infection. A total of 136 cases were identified from a large outbreak over 20 months implicating UK patients, Spanish patients. After epidemiological investigation, eggs consumption was suspected to be at the origin of the outbreak. Additional selected clinical sequences from GenomeTrakr were also included in the analysis.

**Figure 3:**
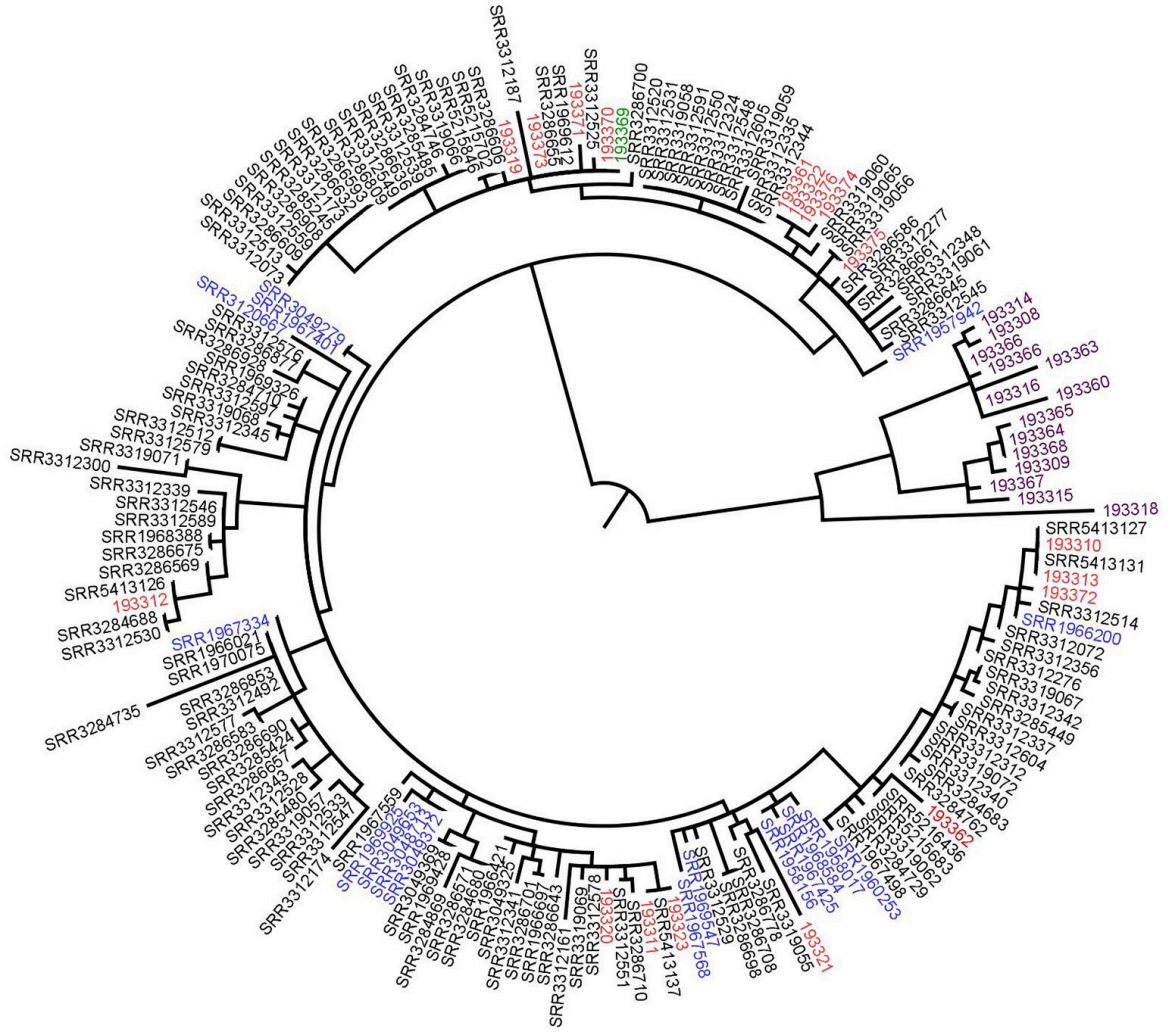
Phylogenetic tree based on SNPs differences from *S*. Enteritidis sequences shared by the authors. Blue: previous UK cases isolates, red: Spanish isolates, black: UK patients' isolates, purple: supplementary data provided by the authors (not linked to the outbreak) and green: the food isolate. Tree was generated with Garli and drawn with Figtree.

We contacted the authors who kindly shared the sequence data. In a first approach, we reproduced the analysis on 177 isolate sequences (176 clinical isolates and one food isolate) using the same *S. enterica* Enteritidis AM933172 genome as reference. At the exception of 14 isolates which had more than 45 SNPs compared to the rest of the isolates, all the isolates had < 20 SNPs differences between them and could be grouped together (Figure 3). With this analysis we obtained the same results as described by Inns and coworkers (Inns et al., 2017).

**Figure 4:**
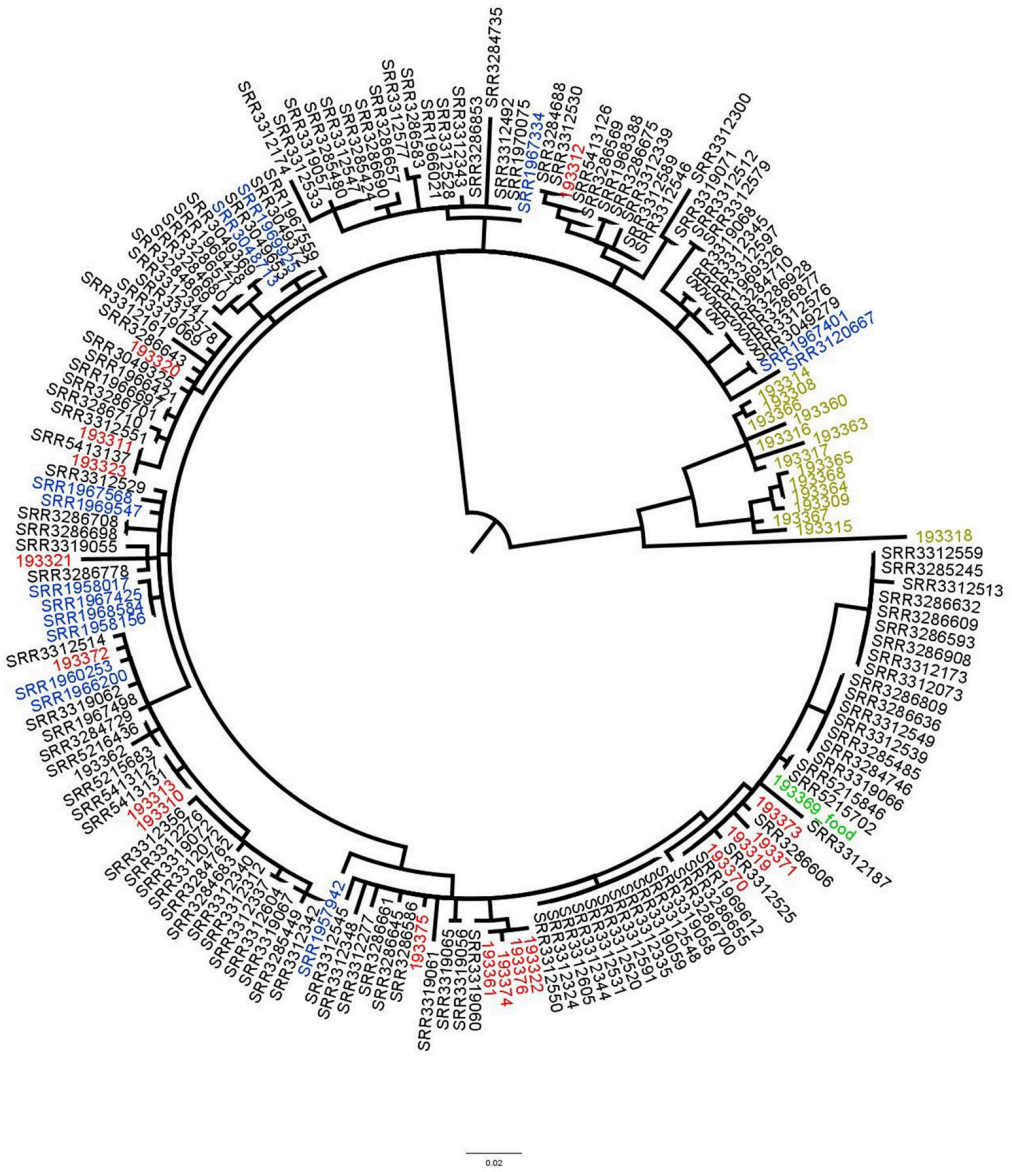
Phylogenetic tree based on SNPs differences from *S*. Enteritidis obtained with the food isolate as reference. Blue: previous UK cases isolates, red: Spanish isolates, black: UK patients' isolates, light green: supplementary data provided by the authors (not linked to the outbreak) and green: the food isolate. Tree was generated with Garli and drawn with Figtree.

With our methodology the food isolate would be the preferred reference to conclude if any patient isolate was related to the food. Therefore, in the second approach, analysis was performed on 177 isolates and the same results were obtained (Figure 4).

### Comparison of hqSNP results using reference genomes obtained from illumina and PacBio sequencing technologies

HqSNP analysis is based on the mapping of Illumina reads on a reference genome. As a good practice, not only the reference genome needs to be genetically related to the genomes analyzed, but also needs to be the most complete as possible, since absent regions will not be analyzed and potential SNPs in these areas would not be detected. Even if there is an advantage of PacBio read length over Illumina, it is less costly and faster to use Illumina data to obtain a reference genome for hqSNP analysis. For that reason, in this study, the impact of the sequencing technology to obtain the reference genome was assessed by using as reference a genome obtained with *de novo* assembled Illumina contigs and a genome obtained with PacBio contigs for *L. monocytogenes* and *S. enterica* strains.

The evaluation was done by the SNP calling on sequences obtained from the stability, repeatability and reproducibility experiments. A total of 22 results obtained with PacBio reference genomes were compared to 22 results obtained with *de novo* assembled Illumina contigs. For all *L. monocytogenes* and *S. enterica* strains, the exact same SNPs were identified when using the PacBio genome or using the Illumina assembled contigs.

## Discussion

The objective of this work was to validate the end-to-end WGS workflow used for source tracking analysis of *L. monocytogenes* and of *S. enterica*. Previously, molecular subtyping tools such as PFGE had been used for source tracking to differentiate related and nonrelated microbial strains to support epidemiological investigations of foodborne outbreaks (Ronholm et al., 2016). Nowadays WGS is replacing conventional molecular subtyping tools due to the higher resolution of the technique (Nadon et al., 2017). The implementation of a new typing technique requires an evaluation of its performance characteristics as previously described (Struelens, 1996). Validation of genomic typing tools is not evident because of the absence of standardized technical procedures, reference materials and criteria for results interpretation. In the current study, WGS applied to source tracking of *L. monocytogenes* and of *S. enterica* was validated using the following performance criteria: stability, repeatability, reproducibility, discriminatory power, and epidemiological concordance (Struelens, 1996). Although the bioinformatics CFSAN SNP Pipeline was previously evaluated on its robustness and accuracy on a dataset of 1,000 *in silico* mutated genomes (Davis et al., 2015), the end-to-end workflow including subculturing, DNA extraction, sequencing and bioinformatics analysis has to the best of our knowledge not been validated. This is the first study addressing the validation of the complete WGS workflow for the application of source tracking. The high resolution of WGS is known to be able to discriminate isolates that were previously shown to have the same PFGE profiles (Moura et al., 2016) or combined PFGE-MLVA (Multi Loci Variable number tandem repeat) profiles (den Bakker et al., 2014; Jackson et al., 2016). Consequently, the differences between genomes identified by WGS need to be trusted and a validation of all steps of the WGS workflow is recommended.

The evaluation of the stability assessed whether the WGS workflow was able to recognize the clonal relatedness of isolates despite the genomic variation that might occur with bacterial replication (Struelens, 1996). We performed a comparison of genomic sequences from five independent colonies from the first subculture and five independent colonies after the tenth subculture. In our experimental setup, few SNPs were observed for the eight *L. monocytogenes* and eight *S*. *enterica* strains evaluated. Allard and coworkers had observed as well one to three substitutions between *S*. Montevideo isolates from different colonies or subcultures (four passages) (Allard et al., 2012). The few SNPs observed in our analysis might be due to biological variations introduced by the subculturing steps and/or to any of the subsequent steps of the WGS workflow (DNA extraction and sequencing). Orsi and coworkers had suggested the possibility of the introduction of SNPs during subculturing of *L. monocytogenes* isolates (Orsi et al., 2008). From the serotypes tested, we did not observe that a specific serotype was more prone to acquire additional SNPs.

The complete WGS workflow generated results with high repeatability and reproducibility which is in concordance with the study of Kozyreva and coworkers (Kozyreva et al., 2017).

We assessed the discriminatory power to ensure that the typing system assigns a different type to two unrelated strains. Unlike conventional typing methods, the ability of WGS to differentiate strains is less of a concern as whole genome data offer the highest possible resolution to elucidate phylogenetic relations (Ronholm et al., 2016). Therefore, the approach to evaluate the discriminatory power was slightly adapted from Struelens and coworkers. in order to illustrate the ability to discriminate unrelated strains while revealing relatedness of close isolates (Struelens, 1996). In the current study, epidemiologically unrelated ATCC or NCTC strains were used. For each unrelated strain, a set of known related sequences obtained from independent cultures of the strain or acquired at different dates were included in the analysis to ensure that the methodology still allowed to identify related isolates. For *L. monocytogenes*, three different serotypes including the serotype commonly associated with food (1/2a) and the serotype commonly related to outbreaks (4b) were evaluated (Nelson et al., 2004). In case of *S. enterica*, three different serovars were evaluated including a previously described clonal serovar such as *S*. Enteritidis (Taylor et al., 2015). For both *L. monocytogenes* and *S. enterica*, the complete WGS workflow allowed to differentiate unrelated strains without losing the ability to identify closely related strains.

The epidemiological concordance evaluated the capability of the CFSAN SNP Pipeline to reproduce results from previously published outbreak investigations using other bioinformatics pipelines. In this exercise, only the bioinformatics analysis was considered as the sequences were obtained from the public database at NCBI or directly from the authors of a published outbreak investigation. We selected one *L. monocytogenes* and one *S. enterica* outbreak investigation in which other bioinformatics pipelines were used. For both studies, agreement between the results obtained with the CFSAN SNP Pipeline and the author's pipelines was observed.

The ideal read mapping analysis would require the usage of a closed genome as reference for SNP calling. The impact of using a high quality Illumina assembly or PacBio assembly genome as reference was evaluated. To the best of our knowledge, no data are reported in the literature to show equivalence. In the present study, we report no impact in terms of SNP calling with these two sequencing technologies, and therefore we conclude that both alternatives to obtain the reference sequence are equivalent in the setup evaluated.

In conclusion, the current study is the first study proposing a validation approach including all steps of the end-to-end WGS workflow. We validated this WGS workflow, for the application of source tracking of *L. monocytogenes* and *S. enterica*, using the stability, repeatability, and reproducibility evaluations. Additionally, the complete WGS workflow was shown to have a high discriminatory power while enabling the ability to show genetic relatedness. For other bacteria, a validation would be required where the same performance criteria, as proposed in the current study, can be used. We believe that this work contributes as a first step to harmonize methods and thus obtain reliable results.

## Author contributions

JG and LB performed the lab experiment steps including the DNA extraction step. CF performed the sequencing of all DNA used in this publication. CN-B, CB, LB, and A-CP designed the experiments described in this publication. A-CP, CN-B, and CB run the bioinformatics analyses. All authors contributed to the writting of the manuscript.

### Conflict of interest statement

The authors declare that the research was conducted in the absence of any commercial or financial relationships that could be construed as a potential conflict of interest.

## Abbreviations

CDC: Center for Disease Control and Prevention
CFSAN: Center for Food Safety and Applied Nutrition
ESCMID: European Society for Clinical Microbiology and Infectious Diseases
ESGEM: European Study Group on Epidemiological Markers
FDA/USFDA: Food and Drug Administration
GMI: Global Microbial Identifier
ILSI: International Life Sciences Institute
ISO: International Organization for Standardization
NCBI: National Center for Biotechnology Information
PHAC: Public Health Agency of Canada
PHE: Public Health England
UK: United Kingdom
USA: United States of America.
